# Large registries, rare outcomes and neutral/inconclusive machine learning results in abdominal wall surgery

**DOI:** 10.3389/jaws.2026.17526

**Published:** 2026-09-21

**Authors:** M. Verdaguer-Tremolosa, Sergi Mojal, V. Rodrigues-Gonçalves, M. P. Martínez-López, M. López-Cano

**Affiliations:** 1 Department of Surgery, UD of Medicine of Vall d’Hebron, Universitat Autònoma de Barcelona, Barcelona, Spain; 2 General and Digestive Surgery Department, Abdominal Wall Surgery Unit, Hospital Universitari Vall d’Hebron, Barcelona, Spain; 3 Institut d’Investigació Biomèdica Sant Pau (IIB-Sant Pau), Barcelona, Spain

**Keywords:** abdominal wall surgery, Artificial intelligence, machine learning, registry, scientific publishing

## Introduction

Artificial intelligence (AI) has become one of the most influential developments in contemporary surgical research, although many surgeons still have limited training in how these tools work and how their results should be interpreted. Machine learning (ML) is increasingly used to develop clinical prediction models, fuelled by the rapid expansion of prospective surgical registries and growing expectations that computational algorithms will improve individualized decision-making. Abdominal wall surgery (AWS) has followed this trend, with several studies exploring AI-based prediction of recurrence, postoperative complications and other clinically relevant outcomes [1–6].

At first glance, national registries containing hundreds of patients appear ideally suited for ML. However, this assumption deserves careful examination. Prediction models do not learn from patient numbers alone, they learn from informative outcome events. In AWS, the outcomes that matter most (mesh infection, recurrence, major surgical site occurrences or severe postoperative morbidity) may be uncommon in certain conditions such as in inguinal hernia repair. Consequently, even apparently large registries may contain relatively little information for predictive modelling.

Our experience analysing the management of inguinoscrotal hernia in the Spanish EVEREG registry illustrates this paradox. Although our cohort included almost 750 inguinoscrotal hernia repairs, postoperative complications occurred in only 52 patients. Multiple algorithms, including random forests, support vector machines, neural networks, k-nearest neighbours and elastic-net regression were explored. We were surprised that none demonstrated clinically meaningful superiority. So, we asked ourselves: are we sometimes asking more from ML than our data can provide? Our experience suggested that the main limitation was not the choice of algorithm, but the amount of information available in the dataset.

We therefore argue that such findings should not be interpreted as negative. They are neutral (or more appropriately, inconclusive) because they identify circumstances in which currently available registry data are insufficient to support robust prediction. This experience is the starting point for this Opinion Article.

## Large registries are not necessarily informative prediction datasets

Prediction studies require more than large cohorts. Effective sample size depends on the number of events, candidate predictors and intended model complexity [7, 8]. A registry may therefore be excellent for epidemiology or quality assurance while remaining insufficiently informative for prediction modelling.

The EVEREG experience highlights this principle. Although the registry represented an excellent national cohort, only a small proportion of patients experienced postoperative complications. ML therefore had relatively few patients with complications from which to learn stable and reproducible patterns. This does not reduce the value of surgical registries, but it reminds us that a good registry is not necessarily a good dataset for prediction.

## Machine learning cannot compensate for limited information

Modern algorithms can detect complex relationships, but they cannot generate biological information that is absent. When clinically relevant outcomes are rare, increasing computational complexity cannot replace genuinely observed events. Importantly, this limitation is not exclusive to ML: if key clinical variables are missing, or if too few outcome events are available, any predictive analysis will be limited (ML or conventional statistical methods). Consequently, different ML algorithms may show similar performance simply because the dataset does not contain enough information to improve prediction further.

Likewise, class imbalance should not be confused with lack of information. Oversampling, undersampling and SMOTE (Synthetic Minority Oversampling Technique) facilitate optimisation but cannot replace new independent patients with real outcome events [9, 10]. Future advances in AWS prediction will therefore depend more on richer clinical variables, improved follow-up and collaborative registries than on increasingly sophisticated balancing techniques.

## Validation, interpretation and clinical usefulness

Scarcity of outcome events also influences validation strategy. Conventional train-test splits may waste valuable information, whereas bootstrap procedures and repeated cross-validation often provide more efficient internal validation when events are limited [11, 12].

Model evaluation should not be judged only by accuracy or AUROC (receiver operating characteristic curve). Calibration, sensitivity, positive predictive value and precision–recall curves frequently provide more meaningful assessments of clinical usefulness in imbalanced datasets [13, 14]. Ultimately, prediction models should be judged by their capacity to improve decision-making rather than by statistical novelty alone.

## Prediction science beyond machine learning

Current evidence indicates that ML does not consistently outperform logistic regression across clinical prediction studies [15–17]. Rather than competing approaches, regression and ML should be viewed as complementary tools. Logistic regression remains valuable because it is simple and easy to interpret, whereas ML models are worthwhile when their additional complexity leads to a clearer improvement in prediction.

Importantly, predictive modelling should not be confused with causal inference. Variables identified as influential by ML are not necessarily causal determinants of postoperative complications. Clinical interpretation therefore remains essential.

## Neutral/inconclusive results are scientifically valuable

Perhaps the principal message of this article is that inconclusive ML analyses deserve greater recognition. Publication bias favours successful algorithms, whereas studies reporting modest performance are often overlooked. Yet these investigations frequently provide equally valuable methodological information because they define the current limits of prediction.

In our opinion, the principal conclusion of the EVEREG experience is not that ML failed. Rather, the registry contained insufficient information to support reliable prediction of uncommon complications. This should not discourage the use of ML, but it should help us design better studies and know when prediction is realistically possible.

## Discussion

The next-generation of AWS registries should focus not only on increasing patient numbers, but also on collecting more complete and clinically relevant information, using consistent definitions, improving follow-up and increasing collaboration between centres.

These developments are likely to contribute more to future prediction performance than replacing one algorithm with another. Frameworks such as TRIPOD + AI and PROBAST + AI provide an excellent methodological foundation for this evolution [18, 19].

AI undoubtedly has an important future in AWS. Nevertheless, prediction performance will always be constrained primarily by the quality and biological richness of the available data. Large registries with rare outcomes remind us that prediction models learn from informative events rather than from patient numbers alone. Neutral machine learning results should therefore not be regarded as failed studies. A model with limited predictive performance should not necessarily be considered a failed study, it may simply show that the available data are not yet sufficient for reliable prediction and help us design better studies in the future.

ML does not simply reveal what algorithms can learn; it also reveals what our registries are currently unable to teach, and it helps us understand the limitations of our own data.
